# Alignment of liquid crystals by polymers with residual amounts of solvents

**DOI:** 10.1038/s41598-017-03243-5

**Published:** 2017-06-08

**Authors:** Alexander M. Parshin, Victor Y. Zyryanov, Vasily F. Shabanov

**Affiliations:** 1 Kirensky Institute of Physics, Federal Research Center KSC SB RAS, Krasnoyarsk, 660036 Russia; 2 Siberian Federal University, Krasnoyarsk, 660041 Russia

## Abstract

The homogeneous nematic layers in liquid crystal cells with treated surfaces are affected by orientational transitions in the electric, magnetic, or temperature fields. The liquid crystal structures formed on solid or liquid surfaces find limited application in identifying the liquid-crystal states by the textures observed in polarized light. The use of surfaces prepared from polymer solutions makes it possible to significantly broaden the range of application of the liquid crystal structures. We investigate the structures with the continuous transformation of the nematic director orientation from radial to planar, which were formed by the polycarbonate surface in the presence of different residual solvents. The structures contained the disclination lines that aligned either by a plate rubbed to provide the homogeneous planar orientation in the LC layer or by a magnetic field applied along the polycarbonate film during the structure formation. The orientational transitions caused by surface treatment, temperature, and electric or magnetic fields in these structures are observed. The comparison of temperature critical distance as well as electric and magnetic coherence lengths with equilibrium length calculated from the expression for the free energy of the nematic is performed. The electro-optical characteristics of the nematic structures are obtained.

## Introduction

Liquid crystals (LCs) in plane-parallel cells confined by solid surfaces have the homogeneous planar or homeotropic orientation or form the structures^1–6^ that can be observed as various textures. Nematic LCs are characterized by the threaded, schlieren, and marbled textures. The textures typical of cholesteric, smectic, or discotic LCs are focal conic, polygonal, fan-shaped, and mosaic. The homogeneous nematic layers formed in cells with treated surfaces are involved in the temperature- and field-induced orientational effects, including the Fredericks or local Fredericks transition^7, 8^. The structures allow the LC states^3^ to be identified via texture observation in polarized light. The use of these structures in the orientational transitions is complicated by the uncontrollable conditions of director field distribution in an LC layer. The controlled conditions can be established in the nematic layers confined by non-solid surfaces with structural formations in the form of isotropic liquid droplets^9, 10^ or domains on the point defect network on the free LC surface^11–13^. However, the necessity in special preparation techniques and existence in the temperature range close to the nematic−isotropic liquid transition make these structures even more difficult to apply.

The use of polymer solution coatings in LC cells allows significant broadening of the range of application of the LC structures, which are molecular formations stable at room temperature. In particular, application of a magnetic field to a nematic LC layer during preparation of a polymer film by evaporation of a solvent from the polyvinylbutyral solution yielded homogeneous structures^14^, which were then studied in the Fredericks transitions. Upon adsorption of nematic molecules on polycarbonate (PC) macromolecules in the presence of a residual solvent, a domain network occurred on the polymer film and continuously transformed to the planar orientation over the nematic layer thickness^15^. In this study, we investigate such structures with the controlled director field distribution in the LC layer and a new nematic texture. The structures are formed and exist at room temperature, undergo the orientational transitions induced by surface treatment, temperature, and electric or magnetic fields, and can be considered as a promising soft organic material with the attractive physical, chemical, and optical properties.

## Materials and Methods

### Materials

**Figure 1:**
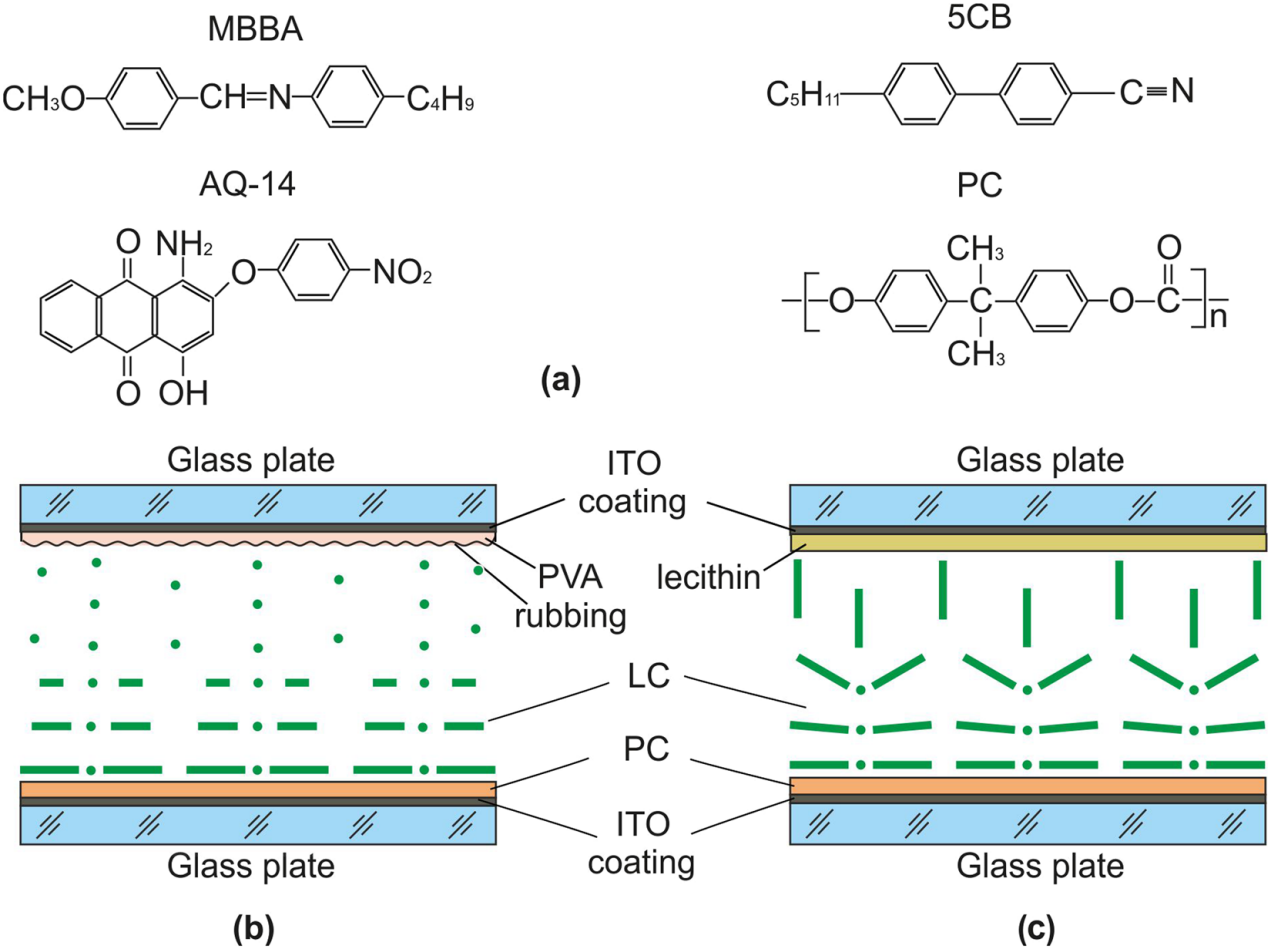
Schematics and arrangement of the materials in cells. (**a**) Chemical structures of the MBBA and 5CB nematics, AQ-14 dye, and polycarbonate (PC). Geometries of (**b**) cell A and (**c**) cell B.

As a polymer, we used granular polycarbonate (PC) synthesized on the basis of Bisphenol A (Sigma-Aldrich Co. LLC). The average molecular weight of PC was 45,000. The grains were dissolved in certain concentrations in one of the three solvents: dichloromethane CH_2_Cl_2_, chloroform CHCl_3_, or pyridine C_5_H_5_N. The dichloromethane stabilized with ~20 ppm of amylene, chloroform stabilized with ~150 ppm of amylene and pyridine, 99% PS (Panreac Quimica S.L.U). As LCs, we used 4 metoxybenzylidene-4′-butylaniline (MBBA) and 4-n-pentyl-4′-cyanobiphenyl (5CB) nematics (both from Merck, Germany) with the phase transition sequences *Cr*–22 °C–*N*–47 °C–*I* and *Cr*–21.5 °C–*N*–35 °C–*I*, where *Cr* is the solid crystal, *N* is the nematic phase, and *I* is the isotropic liquid. In the experiments with colored LC, the 5CB and MBBA nematics were doped with an AQ-14 anthraquinone dichroic dye (0.3 wt.%). The absorption band maximum of the dye was at the wavelength λ_max_ = 0.522 µm. The chemical structures of 5CB, MBBA, AQ-14, and PC are shown in Fig. 1(a).

### Methods

The LC structures were formed under interaction of nematics with the PC surfaces by evaporates of residual solvent prom the polymer film. The preparation conditions are given in Table 1. The polymer solution was deposited onto a substrate by centrifuging and dried at a temperature of *t* = 24 °C for 1 min or annealed in a furnace at temperatures of *t*_a_ = 50 or 120 °C for 15 min. At the solvent evaporation for several seconds with average evaporating rate 40%/min, a PC film formed on the substrate. The film thickness measured using a Filmetric F50-UVX thickness meter (USA) was found to be from 100 nm to several microns. The nematics were deposited onto the polymer film at a temperature of *t*_d_ = 24 or 30 °C.

**Table 1:** The preparation conditions and types of nematic textures on the polymer surface.

Sample no.	Poly mer (wt.%)	Solwent (wt.%)			Liquid crystal		Drying temperature (°C)	Deposition temperapture (°C)	Shaping time (min)	Texture
	PC	CH_2_Cl_2_	CHCl_3_	C_5_H_5_N	MBBA	5CB	t_a_	t_d_	τ	
1	0.2	99.8			٧		24	24	1	t
2	0.2	99.8				٧	24	24	1	g
3	0.6	99.4			٧		24	24	1	g
4	0.6	99.4				٧	24	24	7	g
5	1			99	٧		24	24	1	g
6	1			99		٧	24	24	1	g
7	1	99			٧		50	24	1	e
8	1	99				٧	50	24	1	e
9	1	99			٧		120	24	1	i
10	1	99				٧	120	24	1	i
11	1	99			٧		24	24	15	p
12	1	99			٧		24	24	12	p
13	1		99			٧	24	24	10	p
14	1		99			٧	24	24	10	p
15	2	98				٧	24	24	25	p
16	2	98			٧		24	24	20	p
17	3	97			٧		24	24	40	p
18	3	97				٧	24	24	35	p
19	3	97			٧		24	30	8	p
20	3	97				٧	24	30	5	p

t – thread-likes texture; g – grain-shaped texture; e – enhangled thread-likes texture; i – island-shaped texture; p – polygon nematic texture.

The nematic textures were studied using a polarized light microscope (Olympus BX51, Japan). Micrographs were done by camera (Micropublisher 3.3.RTR, Canada). The temperature for the samples preparation and investigations were controlled up ±0.1 °C (Linkam LTS120, UK). For alignment disclination lines the dc magnetic field *H* ^*^ up to 20 kOe was applied to the cell during the formation of the structure and was generated by an electromagnet.

To obtain the electro-optical and volt-contrast characteristics, two types of LC cells were formed on the basis of sample 15 (Table 1) with two plates. In both cells, the bottom substrate was a glass plate coated with a conducting ITO layer and PC film. Two teflon spacers and a top glass plate with the ITO coating were placed on the film. In the first-type cell (cell A), the top plate was coated with a polyvinyl alcohol (PVA) film and rubbed using a HOLMARC HO-IAD-BTR-O1 machine to specify the planar LC alignment. In the second-type cell (cell B), the top plate was coated with the 1-% lecithin solution to obtain the homeotropic LC alignment. In both cells, the LC layer thickness was δ = 30 µm. The cells were placed at the path of the beam of an R-39727 He-Ne laser (Newport, USA) with a wavelength of λ = 0.633 µm. The 1 kHz voltage *U* from the generator was applied to the LC cell perpendicular to the surfaces and slowly scanned.

## Results and Discussion

### Nematic textures

**Figure 2:**
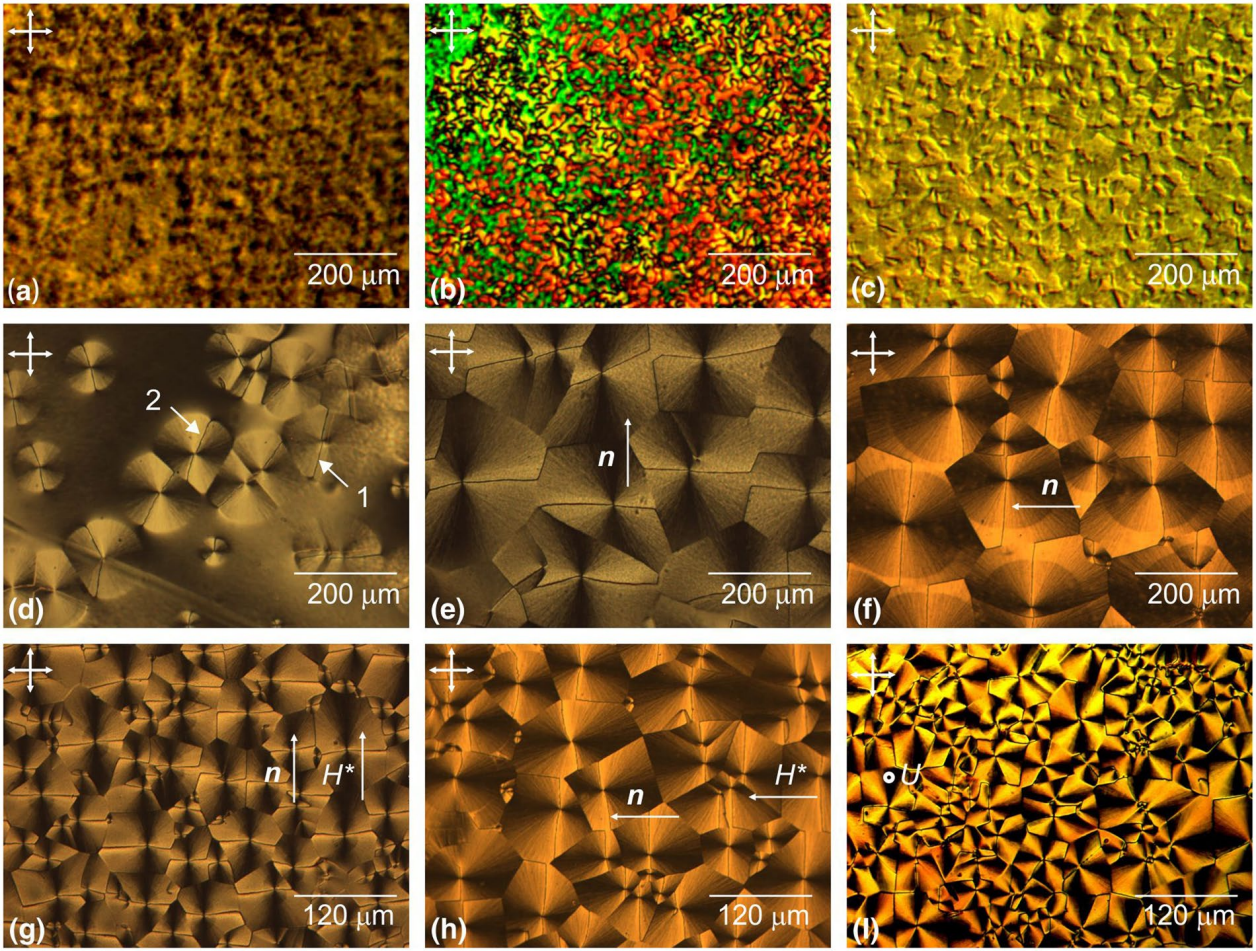
Polarizing optical microphotographs of nematic textures. (**a**) Sample 7: 1% of PC, *t*_a_ = 50°C, *t*_d_ = 24 °C, and τ = 1 min. (**b**) Sample 8: 1% of PC, *t*_a_ = 50°C, *t*_d_ = 24 °C, and τ = 1 min. (**c**) Sample 10: 1% of PC, *t*_a_ = 120°C, *t*_d_ = 24 °C, and τ = 1 min. (**d**) Sample 12: 1% of PC, *t*_a_ = 24 °C, *t*_d_ = 24 °C, and τ = 7 min. (**e**) Sample 16: 2% of PC, *t*_a_ = 24 °C, *t*_d_ = 24 °C, and τ = 20 min. (**f**) Sample 20: 3% of PC, *t*_a_ = 24 °C, *t*_d_ = 30°C, τ = 5 min. (**g**) Sample 16: 2% of PC, *t*_a_ = 24 °C, *t*_d_ = 24 °C, and τ = 20 min in a magnetic field of *H* ^*^ = 20 kOe applied in the PC film plane during the texture formation. (**h**) Sample 18: 2% of PC, *t*_a_ = 24 °C, *t*_d_ = 24 °C, τ = 35 min. (**i**) The “nematic fan texture” obtained from the “nematic polygonal texture” by the electric voltage of *U* = 8.5 V applied perpendicular to the 30-µm-thick LC layer. The director of nematics is **n**. Arrows show the polarization direction.

**Figure 3:**
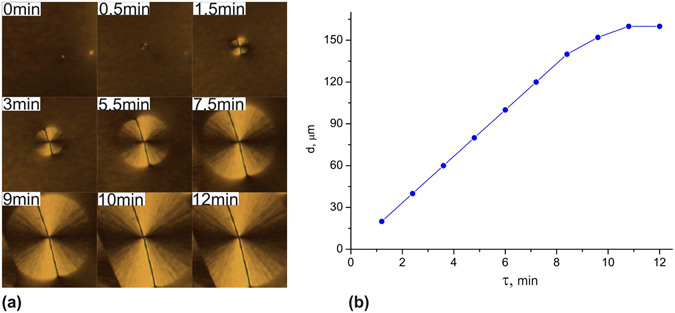
Domain grows. Sample 12: 1% of PC, *t*_a_ = 24 °C, *t*_d_ = 24 °C, and τ = 0–12 min. (**a**) Micrographs of single domain. (**b**) Diameter *d* of single domain vs. τ.

Figure 2 shows the textures obtained under different preparation conditions. At low (<0.6 wt.%) polymer concentrations and, correspondingly, high (>99.4 wt.%) solvent concentrations, the nematics deposited at a temperature of *t*_d_ = 24 °C onto a polymer film dried at the same temperature, form the schlieren, threaded, or “grain-shaped” texture (Fig. 2(a)). The grain-shaped and “entangled thread-like” textures (Fig. 2(b)) are formed also upon annealing of the polymer film at a temperature of *t*_a_ = 50 °C. After annealing at 120 °C for 15 min, the island texture is formed (Fig. 2(c)). The texture formed in the nematics at polymer concentrations over 0.6 wt.% and, correspondingly, CH_2_Cl_2_ or CHCl_3_ solvent concentrations below 99.4 wt.% at *t*_a_ = 24 °C and *t*_d_ = 24 °C resembles a polygonal texture observed in smectics. When the C_5_H_5_N solvent was used, the *nematic polygon texture* did not form at any preparation conditions. It should be noted that the textures shown in Fig. 2(a–c) occur in no more than 1 min after nematic deposition onto the PC film, while the nematic polygonal texture formation takes a long time during the domain growth from nuclei (Fig. 2(d)). The nuclei spontaneously arise on the PC film and increase proportionally to growth time τ, which depends on the polymer and solvent concentrations and temperature *t*_d_ of LC deposition onto the polymer film. At PC concentrations of up to 1 wt.%, the entire domain ensemble forms for τ ≈ 10–15 min at *t*_a_ = 24 °C and *t*_d_ = 24 °C and the average domain size attains *d* ≈ 70–150 µm (Fig. 3). An increase in the PC concentration to 3 wt.% leads to an increase in the growth time to τ ≈ 30–40 min and in the domain size to *d* ≈ 150–200 µm. The dependence of the domain size on the PC concentration is caused by the physicochemical processes occurring at the PC/LC interface. Owing to the extinction, the nematic pulls the residual solvent from the polymer film to its boundary, where the mobility of polymer chains increases and the interaction between PC macromolecules and nematic molecules is intensified. The residual solvent concentration in the PC film determines the frequency of formation of nuclei and their growth slows down when domains touch one another. At the high (over 3 wt.%) polymer concentrations, domains coarsen (*d* ≈ 200 µm); however, the low mobility of polymer chains leads to nonhomogeneous filling of the polymer film surface; i.e., the polymer material appears insufficient to form a high-quality polygonal nematic texture.

An individual domain of polygonal texture can be observed in crossed polarizers as a region with double extinction bands. The domain is divided in two equal or unequal parts by a disclination line passing through its center, which is almost straight along the domain radius. At a certain position of the polarizers, the disclination lines can be seen as thin dark lines (*1* in Fig. 2(d)) or broad bright lines (*2* in Fig. 2(d)). Under synchronous rotation of crossed polarizers, the thin dark lines broaden to *b* ~ 10 µm and brighten. If, during the polygon texture formation, we cover the PC film by a glass plate rubbed to provide the planar orientation in the LC layer, the disclination lines will tend to align perpendicular to the rubbing direction. The degree of their alignment depends on nematic layer thickness ξ. At ξ ≈ 10 µm, only a small part of the disclination lines will be aligned, while at ξ ≈ 2 µm, all the lines will be oriented (Fig. 2(e,f)). Their orientation is similar to that obtained in magnetic field *H* ^*^ applied along the PC film during the structure formation. At *H* ^*^ = 2.5 kOe, only a part of the disclination lines will be aligned. At *H* ^*^ = 20 kOe, we can observe the total alignment of the disclination lines perpendicular to *H* ^*^ (Fig. 2(g,h). In both cases, the director ***n*** of the bulk LC layer tends to align perpendicular to the disclination lines.

If we cover the polygon texture by a glass plate treated in surfactants to provide the homeotropic orientation, then at an LC layer thickness of ξ ≈ 3 µm, the nematic polygonal texture will transform to the texture resembling the fan texture of cholesterics, smectics, or discotics. Similar transformation of the nematic polygonal texture to the *nematic fan texture* (Fig. 2(i)) occurs under the action of temperature or an electric or magnetic field applied perpendicular to the nematic layer. The temperature transition in MBBA and 5CB nematics starts at a temperature of *t*_r_ = *t* − *t*_NI_ = 5.4 °C, where *t*_NI_ is the temperature of the nematic–isotropic liquid transition. During the transition, the sequential change in the domain color from yellow to black at *t*_r_ = *t*_NI_ is observed in crossed polarizers. The transition in the nematic layer with a thickness of ξ ~ 30 µm in an electric field occurred at a threshold voltage of *U*_th_ = 1.6 V and a magnetic field of *H*_th_ = 1.5 kOe and was also accompanied by the texture color change. At *U* ∼ 60 V, a dark field image was established in the LC cell.

**Figure 4:**
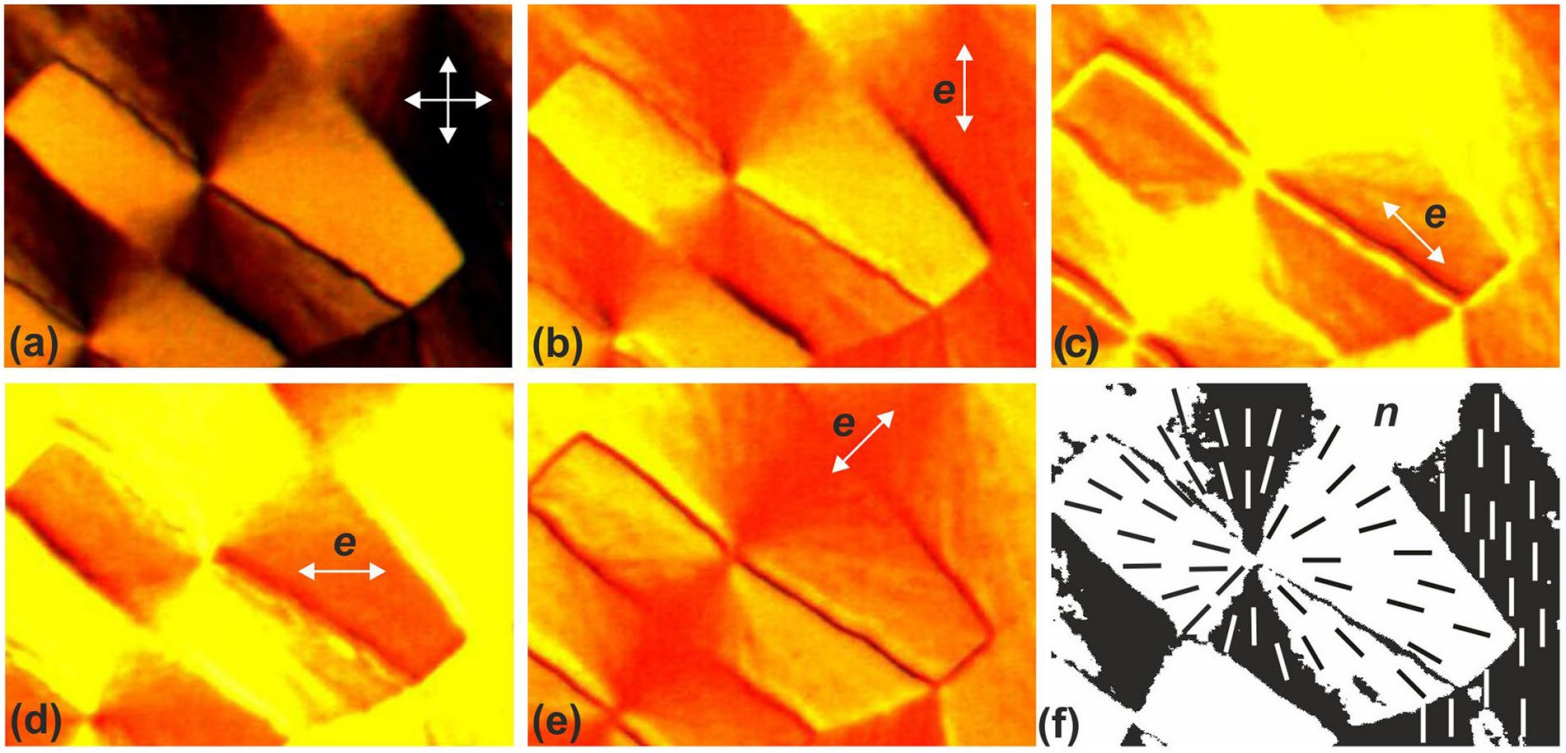
Microphotographs of the dye-doped 5CB domain at certain angles of light polarization *e*. (**a**) In the crossed polarizers; (**b**) 0°;(**c**) 45°; (**d**) 90°; (**e**) −45°. (**f**) The director field configuration in the surface of domain.

An individual domain of nematic fan texture is observed in crossed polarizers as a region with quadruple extinction bands (Fig. 4(a)). A point defect at the domain center has a force of *m* = +1, since under synchronous rotation of crossed polarizers, the band rotation follows the light polarization directions and under sample rotation, it follows the direction opposite to the sample rotation. In the LC doped with a dye, the change in director ***n*** can be followed by the dye color change in one polarizer (Fig. 4(b–e)). The brightest color is observed when the light polarization vector ***e*** coincides with the long axes of dye molecules and, consequently, with the nematic director ***n*** and the disclination line with length *l* is maximally colored at ***e*** ⊥ *l* and brightens at ***e*** || *l*. This investigation shows that in the LC layer adjacent to the PC film, the director field configuration with three structural elements forms. During the growth, radial structure R of the nematic forms on the PC surface (Fig. 4(f)).

The disclination line is a wall localized at the surface and fixed to the surface disclination line (SDL) with length *l* similar to domain diameter *d* and width *b* at which the homogeneous distribution of director ***n*** perpendicular to *l* is implemented. Under normal conditions, structure R would be unstable and the director would “escape” along the third dimension near the domain center^16^. However, the presence of the SDL stabilizes structure R. Owing to adsorption of LC molecules on polymer chains, the R and SDL configurations are localized at the PC surface and a planar or homeotropic bulk layer with director ***n*** parallel or perpendicular to the surface forms over them. Different positions of ***n*** on the R and SDL formations lead to the existence of different stable nematic structures. When ***n*** on structure R makes an angle with the polarization directions of light transmitted through polarizer ***e***_p_ or analyzer ***e***_a_, the SDL can be seen as a narrow dark line. When ***n*** on SDL is close to ***e***_p_ or ***e***_a_, the SDL remains narrow, but structure R can be seen as an area with double extinction bands. If the planar layer is superimposed on structure R, the area with double extinction bands is observed in crossed polarizers. If ***n*** on SDL is close to ***n*** in the bulk of the LC layer, as well as to ***e***_a_ or ***e***_p_, the SDL can be seen as a narrow dark line. When ***n*** on SDL makes an angle of π/2 with the orientation of LC molecules far from the PC film surface, the SDL is observed as a broad bright line.

### Structures of the nematic domains

**Figure 5:**
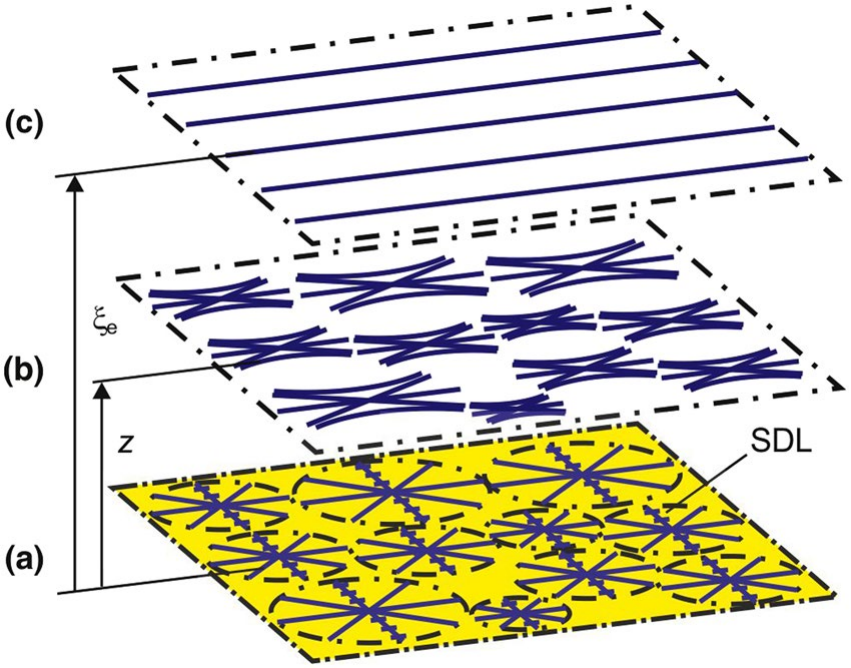
Schematic of the radial-planar structure: on polycarbonate surface. (**a**) At the PC film; (**b**) at a distance *z* from the surface; (**c**) at equilibrium length ξ_*e*_.

Figure 5 shows a schematic of the radial-planar (RP) structure with the SDL. Radial structure R with the SDL is concentrated on the polymer surface and continuously transforms to planar structure P at equilibrium distance ξ_e_ from the surface. Taking into account averaged elasticity constant *K*, we can express the free energy of the LC in volume *V* as^7^

1 $$F=\frac{1}{2\,}K\mathop{\int }\limits_{v}[{(\nabla \cdot {\boldsymbol{n}})}^{2}+{(\nabla \times {\boldsymbol{n}})}^{2}]dV.$$

For structure RP, the components of director ***n*** in the cylindrical system of coordinates *O*ρφ*z* are *n*_ρ_ = −cosψ, ${n}_{{\rm{\phi }}}=\,\cos \,{\rm{\psi }}$, and *n*_*z*_ = 0, where ψ is the angle between director ***n*** and polar radius ρ. The angle of deviation of molecules from ρ on the surface with the molecular adsorption is ψ = 0 at *z* = 0 and ψ = φ at the distance *z* = ξ. Assuming the local distortion of the director field to decay exponentially from the surface^7^ (ψ = (1–exp (–*z*/ξ)) φ and performing integration within *b*/cosφ ≤ ρ ≤ *r*, 0 ≤ φ ≤ 2π − 4·asin(b/ρ), 0 ≤ *z* ≤ ∞, obtain

2 $${F}_{1}=\frac{1}{2\,}K[\pi \,\mathrm{ln}(\frac{2r}{b})\xi +[\pi (\pi -3)+12]\frac{{r}^{2}}{24\xi }+\pi (\pi -4)\frac{r}{4}]+{F}_{l},$$

where *r* is the domain radius, *b* is the SDL width, and *F*_l_ is the SDL energy. Minimization of (2) yields the equilibrium ξ_*e*_ value

3 $${\xi }_{e}={\{\frac{[\pi (\pi -3)+12]}{24\pi \mathrm{ln}(\frac{2r}{b})}\}}^{\frac{1}{2}}r.$$

The SDL is oriented by the rubbed plate or magnetic field *H* ^*^ during the structure formation via oriented molecules of the bulk LC layer. The LC molecules in the SDL are fixed to *l* due to adsorption and tend to rotate cooperatively under the action of molecular forces. In this case, the torque occurs that rotate the SDL perpendicular to the director of the bulk LC layer. The torque value depends on the mobility of polymer chains and the critical equilibrium length, which, as experiments show, is ξ_c_ ≈ 2 µm. The magnetic coherence length^7^ at a field of *H* ^*^ = 20 kOe, a magnetic susceptibility anisotropy of Δχ = 1.16 · 10^−7^, and an elasticity constant of *K* = 6 · 10^−7^ dyn^17^ is also ξ_H_ = 1/*H* ^*^(*K*/Δχ) ≈ 2 µm.

The transition from the nematic polygonal to nematic fan texture can occur when equilibrium length ξ_*e*_ is equal to the coherence length of the external field. Under the assumption of different temperature dependences of structures R and SDL, their intersection at critical distance ξ_t_ from the surface leads to the temperature transition^8^ at ξ_t_ = ξ_*e*_. Approaching of the electric (ξ_E_) or magnetic (ξ_H_) coherence lengths to equilibrium length ξ_*e*_ facilitates the orientational transition in an electric or magnetic field. Substituting the average domain radius *r* ≈ 55 µm and *b* ≈ 10 µm (Fig. 2(g)) in Eq. (3), we obtain a value of ξ_*e*_ ≈ 15 µm, which is consistent with ξ_H_ = 1/*H* (*K*/Δχ) ≈ 15 µm at an experimental value of *H* = 1.5 kOe. At the same time, it is difficult to estimate the ξ_t_ and ξ_E_ values because of the strong electric field nonuniformity in the LC with the strained transition layer of length ξ_*e*_.

### Electro-optical characteristics of the nematic structures

**Figure 6:**
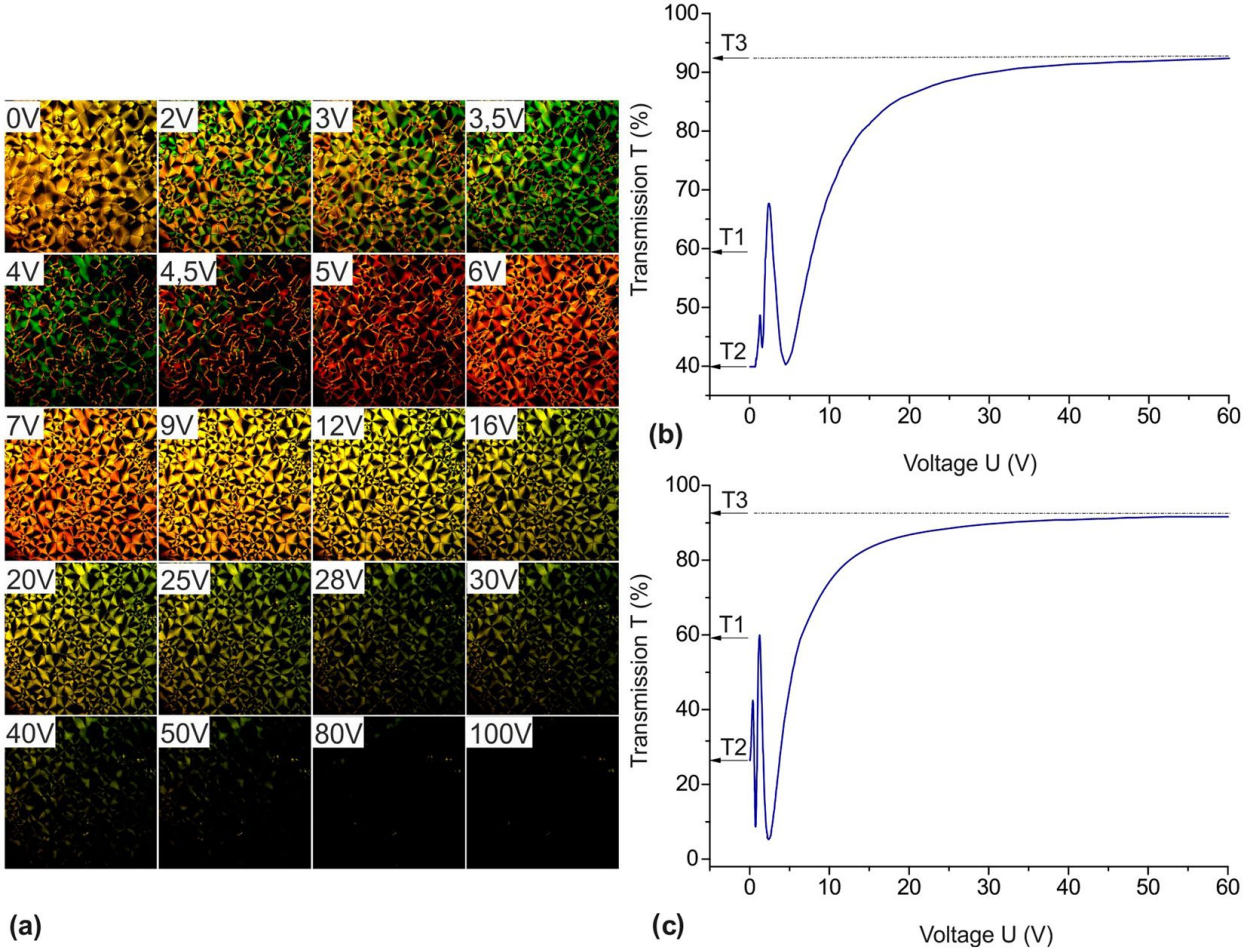
The transition from the nematic polygonal to nematic fan texture. (**a**) The micrographs of domains ensemble at various values *U*. (**b**,**c**) Monochromatic light transmission *T* vs. electric voltage *U*. Laser radiation was transmitted through a cell formed on the basis of sample 15 with the top plate: (**b**) rubbed; (**c**) treated by surfactants.

Figure 6 shows the volt-contrast characteristics *T* (*U*) for structure RP obtained using monochromatic laser radiation in the absence of polarizers. In cell A, for time τ of the structure formation, transmittance *T* decreases from a value of *T*_1_ ≈ 60% corresponding to scattering of the nonhomogeneous structure with the threaded or schlieren texture to a value of *T*_2_ ≈ 40% corresponding to scattering of structure RP. Then, if we apply a field of *H* ^*^ = 20 kOe along the rubbing direction during the structure formation, transmittance *T* will increase to a value of *T*_1_^*^ ≈ 90%, which then decreases to *T*_2_ due to the scattering for time τ. We observed the monotonic variation in *T* from *T*_2_ to *T*_3_, which corresponds to the transition of structure RP to the homeotropic state at a voltage of *U* > 30 V. The volt-contrast characteristic *T* (*U*) is accompanied by interference maxima and minima, which can be attributed to the inhomogeneities caused by structure R. The obtained optical characteristics are comparable with those of the PDLC structures^18, 19^ used in devices based on the light scattering phenomenon. The significant variations in *T* observed in cell B evidence for the stability of structure RP, which remains unchanged at ξ_H_ ≥ ξ_*e*_ even at the competing effect of the top plate in the LC cell.

**Figure 7:**
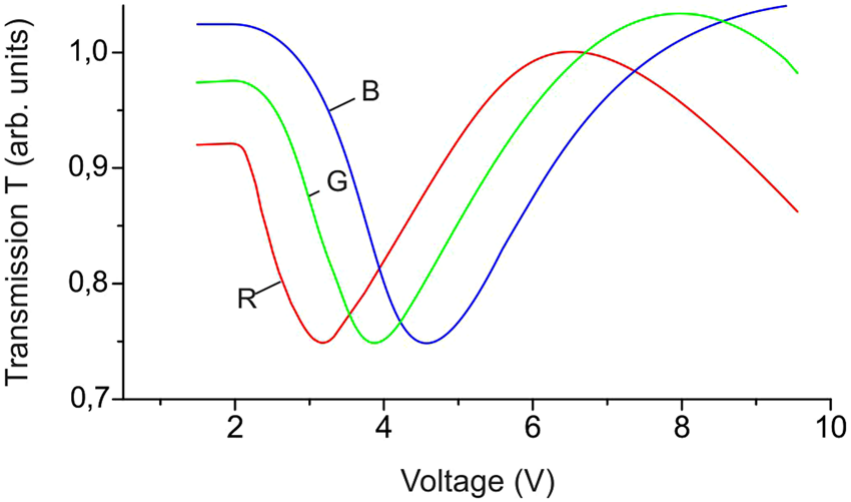
White light transmission *T* of the LC layer vs. electric voltage *U*. The light was transmitted through a cell formed on the basis of sample 15 using red (R), green (G), and blue (B) filters in crossed polarizers.

Figure 7 shows the tricolor characteristics of transmittance of cell A as a function of electric field. To obtain these characteristics, the cell was places between crossed polarizers and illuminated by normally incident white light transmitted through a red (R), green (G), or blue (B) filter with wavelengths of λ(R) = 700, λ(G) = 520, and λ(B) = 452 nm, respectively. It can be seen in Fig. 7 that there is a potential difference between extrema of curves R, G, and B, which is comparable with the differences obtained for hybrid-aligned nematic cells^20^ or cells with color filters^21^ used in multicolor LC displays.

## Conclusion

Thus, the textures of 5CB and MBBA nematics formed by the polycarbonate surface in the presence of CH_2_Cl_2_, CHCl_3_ or C_5_H_5_N were observed as new “grain-shaped”, “entangled thread-like”, and “polygonal” nematic textures. The most attractive is the “polygonal” nematic texture, which has the radial-planar structure consisting of an ensemble of domains grown from nuclei. Each domain is divided in two equal or unequal parts by a disclination line with the homogeneous molecular layer, which ensures the stability of the radial configuration via preventing the director “escape” along the third dimension. The disclination lines are aligned by rubbing a plate to provide the planar orientation in the LC layer with a thickness of ξ ≈ 2 µm and by a magnetic field of *H* ^*^ = 20 kOe applied along the polycarbonate film during structure formation. If we cover the radial-planar structure by a plate treated in surfactants to provide the homeotropic orientation, then at an LC layer thickness of ξ ≈ 3 µm, this structure transforms to the radial-homeotropic structure. The similar transformation in the nematic layer with a thickness of ξ ~ 30 µm occurs at a temperature of *t*_r_ = *t* − *t*_NI_ = 5.4 °C, as well as in an electric field at a threshold voltage of *U*_th_ = 1.6 V or a magnetic field of *H* = 1.5 kOe, which are applied perpendicular to the nematic layer. The radial-planar structure is characterized by the equilibrium distance from the polymer surface ξ_*e*_. When the critical temperature length ξ_t_ and electric (ξ_E_) or magnetic (ξ_H_) coherence lengths approach ξ_*e*_, the orientational transition from the radial-planar to homeotropic-planar structure occurs. The volt-contrast characteristics *T* (*U*) are accompanied by interference maxima and minima. In the electro-optical characteristics of light transmittance *T* of the radial-planar structure as a function of voltage *U*, there is a potential difference between extrema of red, green, and blue light. These phenomena can be used in light-scattering devices and displays.
